# Evolution of artificial intelligence in breast cancer pathology: a bibliometric analysis

**DOI:** 10.1007/s12672-026-05388-0

**Published:** 2026-06-18

**Authors:** Junjie Li, Lei He, Jun Du, Di Cui, Zhengxian Chen, Dongge Liu

**Affiliations:** Department of Pathology, Beijing Hospital, National Center for Gerontology; National Clinical Research Center for Gerontology; The Key Laboratory of Geriatrics of NHC; Institute of Geriatric Medicine, Chinese Academy of Medical Sciences, Beijing, P.R. China

**Keywords:** Breast cancer, Artificial intelligence, Deep learning, Pathology, Bibliometric analysis

## Abstract

**Background:**

Breast cancer is the most common malignant tumor affecting women, and pathology serves as the primary method for its diagnosis. In recent years, artificial intelligence (AI) has increasingly been applied to the pathological diagnosis of breast cancer. This study, therefore, aims to conduct a bibliometric analysis of the literature on artificial intelligence in breast cancer pathology (AIBCP) to map the research landscape, identify key trends, and highlight emerging directions.

**Methods:**

A total of 1089 relevant publications from 2011 to 2025 were retrieved from the Web of Science (WOS) database. Bibliometric analysis was conducted using bibliometric VOSviewer, and CiteSpace, while BERTopic modeling was applied to extract semantic topics from abstracts. The study examined publication trends, geographical and institutional contributions, author and journal profiles, citation patterns, references, as well as keywords and abstract-based topics.

**Results:**

Research on AIBCP has grown substantially since 2017, with 2025 recording the highest number of publications. The majority of them are original articles (70.25%). The leading contributing countries and regions are the USA, China, India, and the European Union, while the University of Toronto emerged as the most productive institution. Key influential authors include Jeroen van der Laak and Nasir Rajpoot. The most cited study focused on deep learning for lymph node metastasis detection. Fourteen distinct research themes were identified, with Topic 0, centered on deep learning for histopathology image analysis, being the dominant topic (53.54%). Other topics included mitosis detection and analysis, tumor microenvironment and prognosis, spatial transcriptomics and gene expression, natural language processing, and multimodal and explainable AI models. Topic analysis reveals a shift from unimodal image analysis towards multimodal “pathomics” and emerging interdisciplinary areas.

**Conclusion:**

The field of AIBCP is rapidly evolving, driven by deep learning-based pathological image analysis and increasingly expanding into comprehensive multimodal and clinical research. Future directions should focus on addressing the AI “black box” problem, to improve interpretability, advancing multimodal approaches, and optimizing human-AI collaboration frameworks. This bibliometric analysis provides a foundational resource to guide researchers and clinicians in navigating and advancing the field.

**Supplementary Information:**

The online version contains supplementary material available at https://doi.org/10.1007/s12672-026-05388-0.

## Introduction

In 2022, breast cancer became the most prevalent tumor among women worldwide and ranked first in cancer-related mortality among females. Statistics indicate that over 2.3 million new cases of female breast cancer were diagnosed globally in 2022, and more than 650,000 deaths were due to the disease [1]. Optimizing cancer screening technologies and implementing accurate early diagnostic methods are critical for reducing breast cancer mortality [2]. Pathology remains the gold standard for distinguishing between benign breast conditions and breast cancer. However, current pathological diagnosis largely relies on pathologists’ visual examination of the lesions under a microscope, which introduces significant subjectivity, limits repeatability, and is both time-consuming and labor-intensive, thereby hindering the accurate diagnosis [3–5].

Artificial intelligence (AI), a technology that simulates human cognition, mainly utilizes machine learning techniques to analyze, recognize, and interpret complex data [6–8]. Since 2012, advances in algorithms and computing power have driven significant progress in machine learning, particularly deep learning, enabling its widespread application across medical fields, such as diagnosis, prognosis prediction, drug development, and genetic analysis [9–12]. In the pathological diagnosis of breast cancer, AI can assist in the segmentation and classification of benign breast and malignant lesions, providing detailed histopathological parameters, including nuclear grade, mitotic activity, axillary lymph node metastasis, and tumor microenvironment [13–16]. Compared with traditional computer-based image measurement, AI can effectively handle the complexity and variability of pathological images, improve the accuracy and efficiency of breast cancer detection, and overcome the limitations of manual visual diagnosis [17, 18].

Although research related to breast cancer is growing at an unprecedented rate, covering a wide range of topics from molecular mechanisms to clinical diagnostic applications [19–21], bibliometric analyses focusing on the topic of artificial intelligence in breast cancer pathology (AIBCP) remain scarce. This is largely due to the growing volume and fragmented nature of the literature. To address this gap, we retrieved relevant articles from the Web of Science (WOS) database and conducted a bibliometric analysis covering the period from 2011 to 2025. This study aims to systematically review research trends, assess the current status of the field, and identify emerging directions for future investigation. The findings of this bibliometric analysis are expected to provide valuable insights for researchers interested in the field of AIBCP. The remainder of this paper is structured as follows: Sect. 2 outlines the bibliometric methods employed, Sect. 3 presents the results of the analysis, Sect. 4 discusses future research directions and the study limitations, and Sect. 5 concludes the paper.

## Methods

### Data collection

Several comprehensive literature retrieval tools are currently available, including Scopus and WOS. Following the recommendations of Donthu et al., we chose to collect bibliometric data from a single database [22]. In this study, WOS served as the data source. Given that the topics analyzed involve multiple disciplines such as oncology, histopathology and computer science, WOS is considered highly suitable for bibliometric analysis. Moreover, compared with databases such as Scopus, WOS is widely recognized for the reliability of its literature and its user-friendly features, which facilitate efficient bibliometric analysis.

Based on our research objectives, three primary keywords were identified: breast cancer, artificial intelligence and pathology. We then expanded these keywords with relevant synonyms to create a comprehensive search string, as follows:

Topic = (“breast cancer” OR “breast carcinoma” OR “cancer of breast” OR “breast tumor” OR “malignant neoplasm of breast” OR “breast malignancy” OR “human mammary cancer”) AND (“artificial intelligence” OR “deep learning” OR “neural networks” OR “machine learning”) AND (“pathology” OR “histopathology” OR “cytopathology”).

**Fig. 1:**
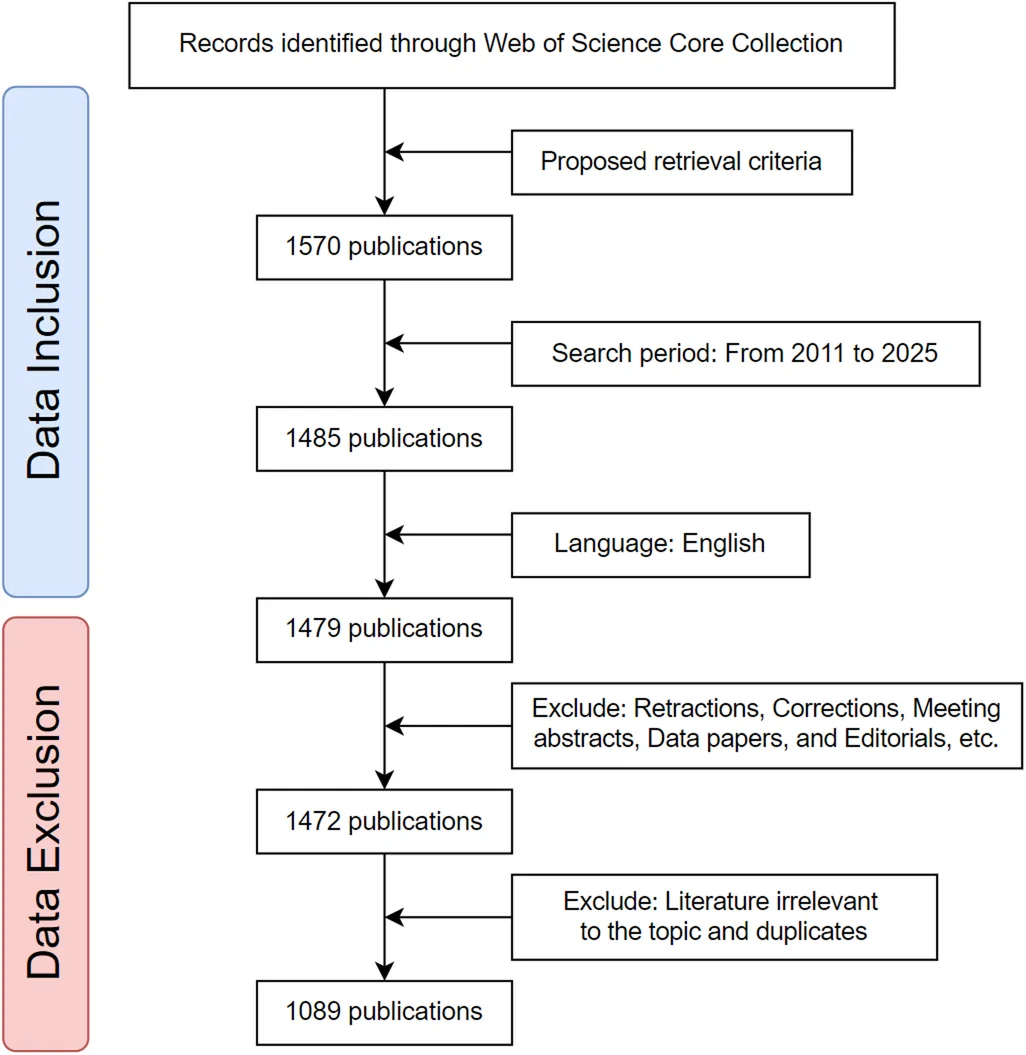
Flowchart of Literature Inclusion and Exclusion. The figure illustrates data inclusion and exclusion process. Initially, 1570 records were identified through the WOS Core Collection using the search criteria. After limiting the search to the period 2011–2025, 1485 records remained. Screening for English-language publications resulted in 1479 records. Subsequently, retractions, corrections, meeting abstracts, data papers, editorials, and other non-target document types were excluded, leaving 1472 records. After further removal of literature irrelated to the topic and duplicates, 1089 studies were ultimately included for subsequent analysis

The literature search was conducted in WOS core collection and on March 12, 2026. We limited the literature search to the period from January 01, 2011 to December 31, 2025. Inclusion criteria comprised English-language original articles, reviews, and conference proceedings related to the search keywords. Excluded were letters, brief communications, retractions, corrections, editorials, book reviews, duplicates, and any publications not relevant to AIBCP. According to these criteria, a total of 1089 documents were identified, with the completeness of each entry ranging from 71.07% to 100% (Supplementary Table 1). Figure 1 illustrates the inclusion and exclusion process. This dataset was subsequently used for the extraction and visual analysis of various information, including countries/regions, institutions, authors, journals, citations and references, keywords, and abstracts.

### Data analysis

Basic statistical analysis of the data was first performed using the pyBibx package [23]. Bibliometric visualization and network analyses were then conducted with specialized tools. VOSviewer (v1.6.20) and Pajek (v6.01) were used to construct and visualize networks among countries/regions, institutions, authors, journals, and cited references [24, 25], revealing key relationships and. Scimago Graphica (v1.0.52) generated geographic maps to depict the spatial distribution of scientific contributions [26], while CiteSpace (v6.4.R1) was employed for keyword analysis [27]. This software facilitated the creation of a keyword co-occurrence network, performed cluster analysis to identify research themes, provided a timeline view to track their development, and applied burst detection to pinpoint emerging trends.

### Topic analysis

To deeply explore the semantic structure and research themes of literature abstracts in the AIBCP field, we used the BERTopic model for topic analysis [28]. First, based on the “all-mpnet-base-v2” pre-trained model, the abstracts were converted into high-dimensional text semantic vectors. Dimensionality reduction was then performed using the UMAP algorithm. Subsequently, the HDBSCAN density clustering algorithm was applied to identify potential thematic clusters. During the topic representation stage, a class-based TF-IDF algorithm combined with topic word diversity control was used to extract representative keywords for each topic. Through parameter sensitivity analysis (Supplementary Table 2), the parameters for the aforementioned algorithms were set as follows: n_neighbors = 15, min_dist = 0.1, n_components = 3, min_cluster_size = 5, min_samples = 1, nr_topics = 15, diversity = 0.3. Upon completion of the modeling, the model’s quality was validated using a combination of metrics. The topic coherence score (c_v) was calculated to assess internal semantic consistency. The topic diversity score was evaluated to ensure effective differentiation between topics. Finally, three experts in the AIBCP field were invited to independently conduct manual scoring for topic interpretability. Additionally, we used the same search strings and inclusion/exclusion criteria to retrieve 1,989 documents from the Scopus database for external validation of the topic.

## Results

### Description of the data

As shown in Table 1, of the 1089 relevant publications, 765 are research articles (70.25%), 111 are review articles (10.19%), and 213 are conference proceedings (19.56%). These publications come from 1688 institutions across 89 countries and regions and involve a total of 5312 authors, averaging 1.30 articles per author. The publications have been cited a total of 30,680 times, yielding an average of 28.17 citations per article. There are 2017 author keywords and 879 keywords plus entries. The dataset includes 28,505 cited references, with an average of 26.18 references per article.

**Table 1:** Summary of the data

Main information	Results
Total Number of Documents	1089
Article	765
Review	111
Proceeding paper	213
Time Span	2011–2025
Average Documents per Year	72.60
Total Number of Countries/Regions	89
Total Number of Institutions	1688
Average Documents per Institution	4.82
Total Number of Authors	5312
Average Documents per Author	1.30
Total Single-Authored Documents	20
Total Multi-Authored Documents	1069
Total Number of Sources	326
Average Documents per Source	2.71
Total Number of Citations	30,680
Average Citations per Author	5.78
Average Citations per Institution	18.18
Average Citations per Document	28.17
Average Citations per Source	87.27
Total Number of References	28,505
Average References per Document	26.18
Total Number of Authors Keywords	2017
Total Number of Authors Keywords Plus	879

### Analysis of publications

**Fig. 2:**
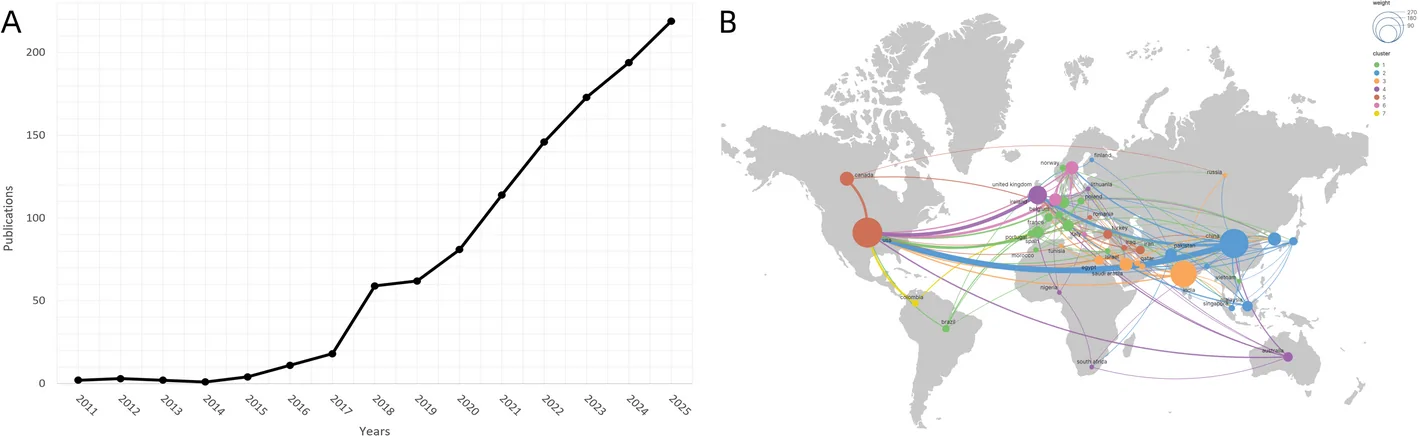
Annual Trends and Global Collaboration of AIBCP. **A **Continuous growth in publication numbers of AIBCP from 2011 to 2025. **B** Global collaboration network map, where node size represents publication volume, link reflects collaboration strength, and colors distinguish research clusters, highlighting the central role of countries such as the United States and China in the collaborative network

The annual number of publications on AIBCP has steadily risen, from 2 articles in 2011 to 219 articles in 2025. Notably, since 2017, the yearly output has increased sharply (Fig. 2A).

Regarding publication by country/region (Supplementary Table 3), the USA leads with 246 articles, followed by China and India with 232 and 191 articles, respectively. The United Kingdom ranks next, with 94 publications, showing a notable gap. Among the top 20 countries/regions, France has the highest average citations per article at 98.850, followed by the Netherlands at 95.238. Analysis based on the number of publications per million population (PPMP) shows that Sweden has the highest number (4.671 PPMP), followed by the Netherlands (2.436 PPMP) and Saudi Arabia (1.590 PPMP). In contrast, although the United States, China, and India lead in terms of total publication volume, their PPMP numbers are relatively low due to their large populations, at 0.754, 0.167, and 0.140 PPMP, respectively. In addition, the data indicate that developed countries with smaller populations (such as Sweden, the Netherlands, and Canada) generally outperform developing countries with larger populations on PPMP. Figure 2B illustrates four major research hubs: the USA, China, India, and the European Union. These countries/regions maintain extensive collaborations with other countries/regions.

**Fig. 3:**
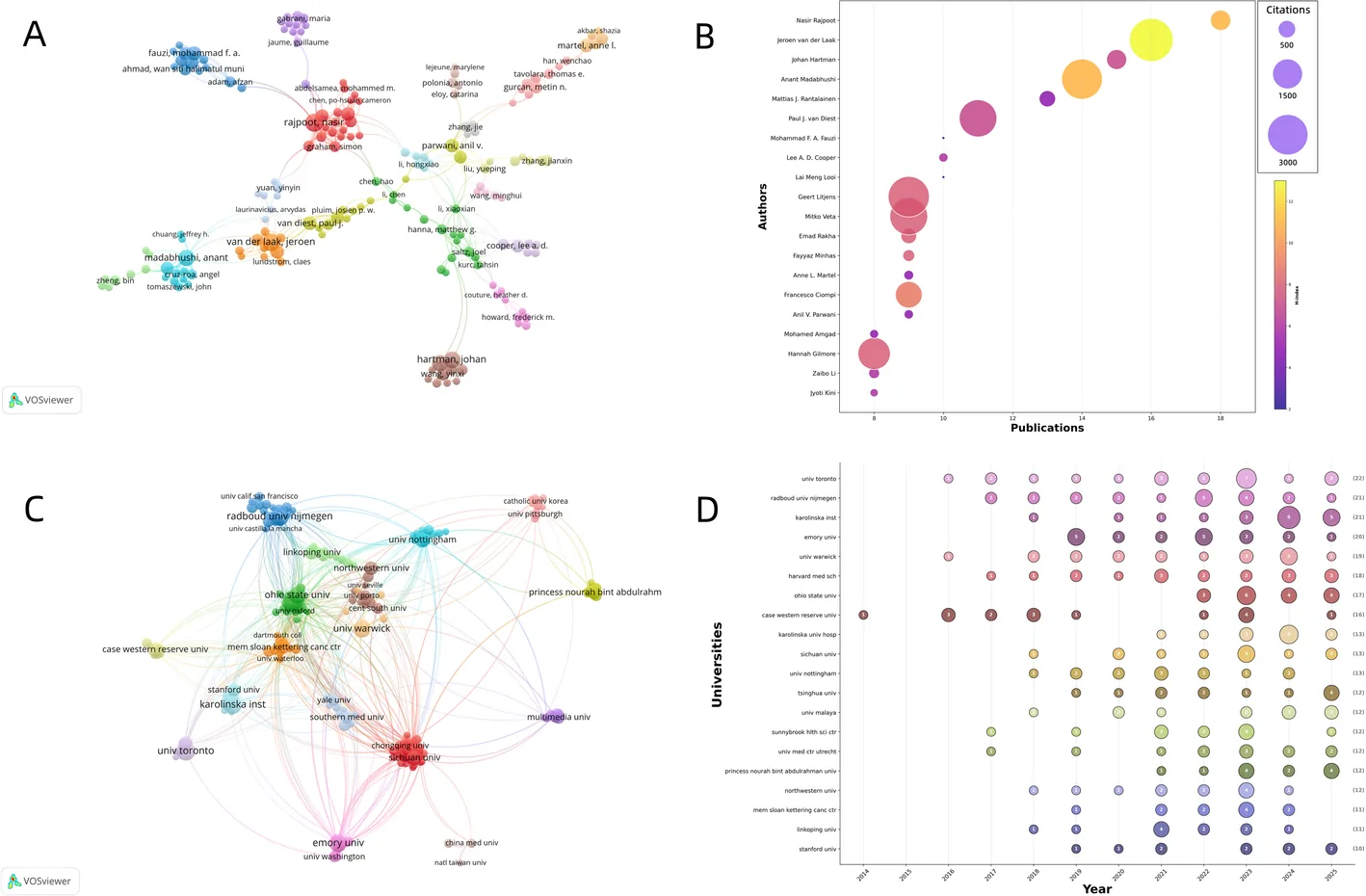
Author and Institution Collaboration in AIBCP. **A** Author collaboration network illustrating core research teams and their cooperative relationships. **B** Bubble chart of prolific authors, with the horizontal axis representing publication volume, the vertical axis listing author names, bubble size indicating citation frequency, and color reflecting H-index. **C **Institutional collaboration network showing the collaborative linkages among major research institutions. **D** Annual publication trends of institutions, where dot size represents publication volume per year

Table 2 lists the top 20 authors, in terms of publication output. Nasir Rajpoot leads with 18 articles, followed by Jeroen van der Laak (*n* = 16) and Johan Hartman (*n* = 15). Regarding average citations, the top five authors are Jeroen van der Laak, Geert Litjens, Anant Madabhushi, Mitko Veta, and Paul J. van Diest. Figure 3A illustrates a co-authorship network, showing distinct author clusters that reflect the formation of multiple closely collaborative research teams within the field. Figure 3B illustrates a positive correlation between the number of AIBCP publications and the local H-index; however, some authors achieve higher H-indices despite similar publication counts, suggesting that both the quality and quantity of papers are important in determining an author’s impact. Institution analysis shows that researchers from the University of Toronto contributed the highest number of publications, with a total of 22 articles. Among the top 20 institutions (Supplementary Table 4), seven are based in the USA. The institutional cooperation network indicates Harvard Medical School has the highest overall collaboration intensity, reflecting the strongest tendency for inter-institutional cooperation (Fig. 3C). Analysis of institutional productivity also reveals that, since 2017, many institutions experienced synchronous growth in their publication output (Fig. 3D).

**Table 2:** Overview of the top 20 authors with the most publications

Authors	Publications	Citations^*^	Avg. pub. year	Avg. citations	Local H-index
Nasir Rajpoot	18	727	2021.778	40.389	11
Jeroen van der Laak	16	3354	2020.688	209.625	13
Johan Hartman	15	707	2022.933	47.133	7
Anant Madabhushi	14	2903	2017.714	207.357	11
Mattias J. Rantalainen	13	473	2023.462	36.385	5
Paul J. van Diest	11	2490	2022.273	226.364	7
Lee A. D. Cooper	10	149	2021.800	14.900	6
Lai-Meng Looi	10	15	2023.500	1.500	2
Mohammad F. A. Fauzi	10	15	2023.500	1.500	2
Geert Litjens	9	2980	2020.556	331.111	8
Mitko Veta	9	2510	2020.556	278.889	8
Emad Rakha	9	429	2021.667	47.667	8
Fayyaz Minhas	9	249	2022.222	27.667	8
Anne L. Martel	9	165	2019.889	18.333	5
Francesco Ciompi	9	1262	2020.333	140.222	9
Anil V. Parwani	9	151	2023.667	16.778	5
Hannah Gilmore	8	1845	2016.500	230.625	8
Zaibo Li	8	209	2023.500	26.125	6
Mohamed Amgad	8	137	2021.250	17.125	5
Jyoti Kini	8	118	2021.875	14.750	6

*Citation counts represent the combined total of self-citations and citations by others

By analyzing the author-institution collaboration networks across different time periods, this study reveals the stable collaborative clusters within the field, centered around a small number of highly productive scholars and connecting multiple institutions. These core authors not only maintain close collaborations within their own institutions but also, through sustained cross-team and cross-regional cooperation, lead the development and validation of key technologies. Institutional collaboration exhibits a hub-and-spoke structure, with institutions such as Radboud University Nijmegen, University of Warwick, and Northwestern University serving as hubs. Through long-term, stable project partnerships and resource sharing, they have constructed a globalized research network. Notably, emerging institutions are gradually integrating into this network through periphery-to-core connections, reflecting the openness and sustainability of the collaborative ecosystem in this field.

**Fig. 4:**
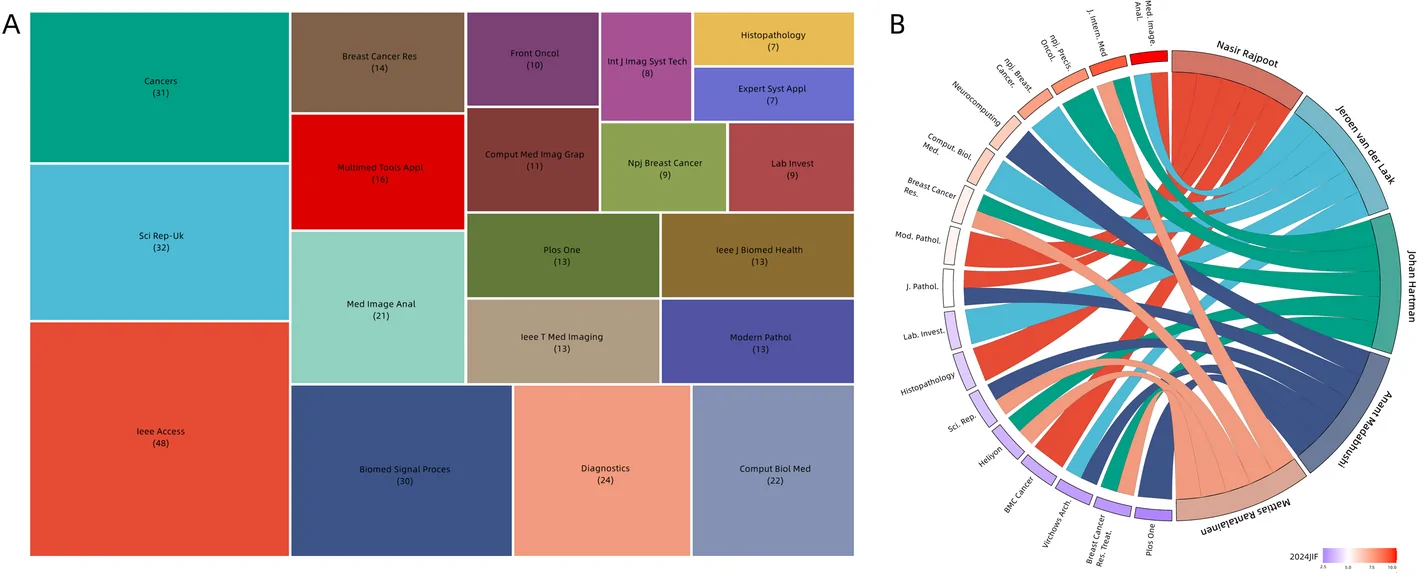
Journal Distribution and Author-Journal Associations in AIBCP. **A** Treemap of journal publication volumes, where the area of each rectangle corresponds to the number of publications per journal, highlighting *IEEE Access*, *Scientific Reports*, and *Cancers* as high-output journals. **B** Chord diagram illustrating author-journal associations. The colored arcs link authors, the journals in which they have published, and the corresponding journals’ 2024 JIF values, visually demonstrating the interaction between high-impact authors and high-JIF journals

Finally, we analyzed publication sources (Table 3). The 1089 papers were published across 326 different sources, averaging 2.71 papers per source. The most prolific journal was “*IEEE Access*”, followed by “*Scientific Reports*” and “*Cancers*” (Fig. 4A). Among the top 20 sources, 13 are JCR Q1 journals, indicating that most research in this field appears in high-impact journals. Figure 4B illustrates the papers of the five most productive authors with the highest publication on AIBCP are largely published in reputable journals.

**Table 3:** Overview of the top 20 journals with the most publications

Sources	2024 JCR Quartile	Publications	Citations	Avg. Pub. Year	Avg. Citations
Ieee Access	Q2	48	841	2023.271	17.521
Sci Rep-Uk	Q1	32	967	2022.375	30.219
Cancers	Q2	31	708	2022.452	22.839
Biomed Signal Proces	Q2	30	323	2023.800	10.767
Diagnostics	Q1	24	247	2023.458	10.292
Comput Biol Med	Q1	22	909	2021.273	41.318
Med Image Anal	Q1	21	1559	2022.143	74.238
Multimed Tools Appl	NA	16	229	2022.438	14.313
Breast Cancer Res	Q1	14	204	2023.357	14.571
Ieee T Med Imaging	Q1	13	1646	2020.308	126.615
Modern Pathol	Q1	13	304	2023.615	23.385
Plos One	Q2	13	199	2022.692	15.308
Ieee J Biomed Health	Q1	13	239	2023.077	18.385
Comput Med Imag Grap	Q1	11	261	2021.455	23.727
Front Oncol	Q2	10	72	2023.200	7.200
Npj Breast Cancer	Q1	9	380	2021.667	42.222
Lab Invest	Q1	9	112	2023.556	12.444
Int J Imag Syst Tech	Q2	8	183	2022.500	22.875
Expert Syst Appl	Q1	7	396	2022.000	56.571
Histopathology	Q1	7	222	2021.714	31.714

### Analysis of citations and references

**Fig. 5:**
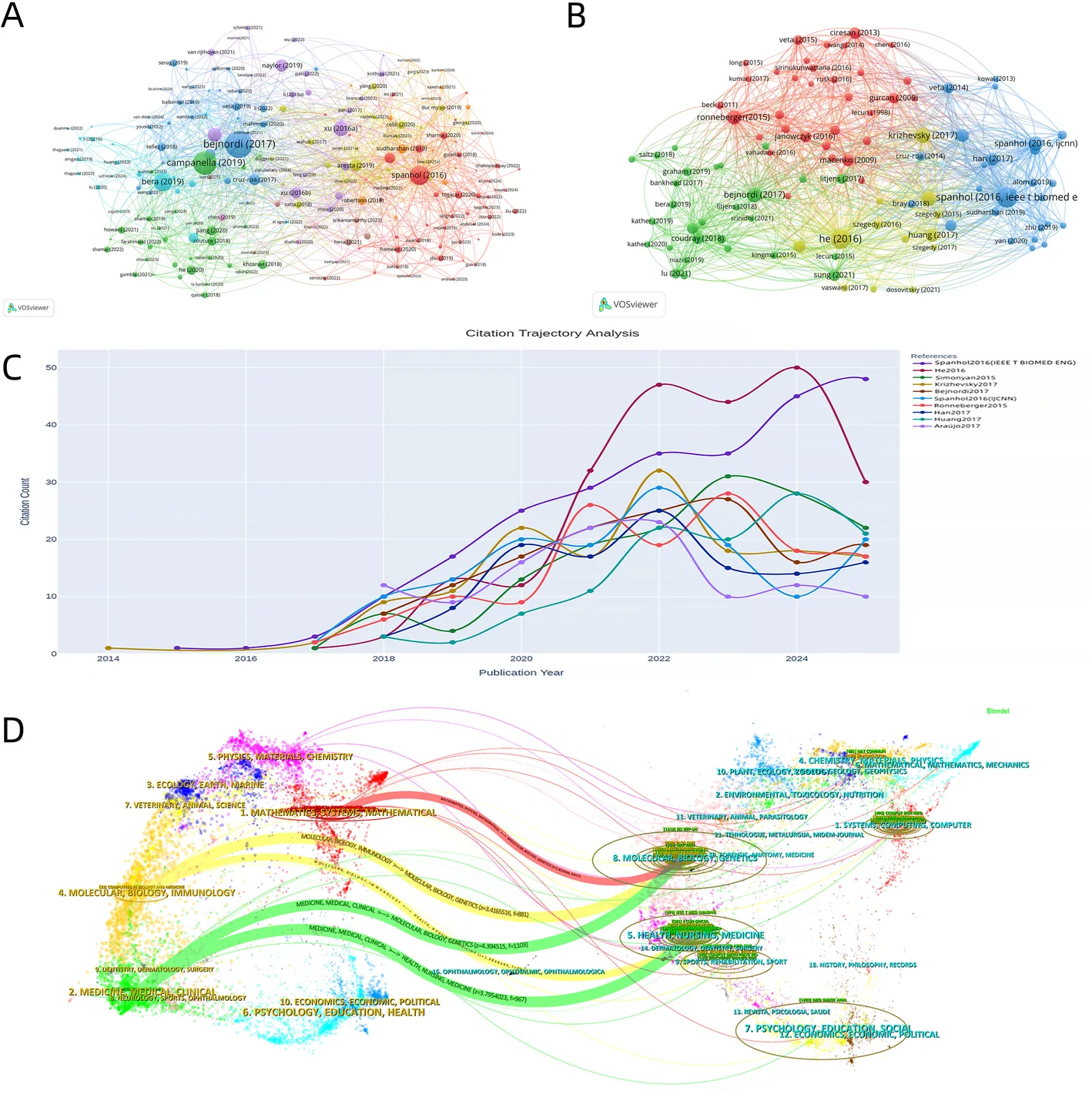
Visualization of Citation and Reference Analysis in AIBCP. **A** Citation network, in which nodes represent individual publications, edges denote citation relationships, and colors correspond to distinct thematic clusters. **B** Reference co-citation network. Each node represents a cited reference, while edges indicate co-citation relationships and their relative strength. **C** Citation trajectory analysis of references, with the horizontal axis indicating year, the vertical axis showing citation counts, and the curves tracking the citation growth of seminal works. **D** Journal overlay plot, with edges indicating inter-journal links and clusters displaying disciplinary structure

Based on the total citation counts provided by WOS, we conducted the citation and references analysis. As shown in Table 4, the most highly cited publication is “Diagnostic Assessment of Deep Learning Algorithms for Detection of Lymph Node Metastases in Women with Breast Cancer” by Babak Ehteshami Bejnordi (2017). Among the top 20 most cited works (Fig. 5A), Bejnordi (2017), Campanella (2019), Spanhol (2016), and Bera (2019) occupy key nodes, potentially acting as bridges or hubs within the citation network.

**Table 4:** Overview of the top 20 publications with the most citations

DOIs	Citations	First author	Year	Type	Sources
https://doi.org/10.1001/jama.2017.14585	2103	Babak Ehteshami Bejnordi	2017	Article	Jama-J Am Med Assoc
https://doi.org/10.1038/s41591-019-0508-1	1581	Gabriele Campanella	2019	Article	Nat Med
https://doi.org/10.1109/TBME.2015.2496264	1031	Fabio Spanhol	2016	Article	Ieee T Bio-Med Eng
https://doi.org/10.1038/s41571-019-0252-y	957	Kaustav Bera	2019	Article	Nat Rev Clin Oncol
https://doi.org/10.1109/TMI.2015.2458702	621	Jun Xu	2016	Article	Ieee T Med Imaging
https://doi.org/10.1038/s41591-021-01343-4	539	Jeroen van der Laak	2021	Review	Nat Med
https://doi.org/10.1186/s13073-021-00968-x	517	Khoa A. Tran	2021	Review	Genome Med
https://doi.org/10.1109/TMI.2018.2865709	433	Peter Naylor	2019	Article	Ieee T Med Imaging
https://doi.org/10.1016/j.media.2019.05.010	406	Guilherme Aresta	2019	Article	Med Image Anal
https://doi.org/10.1038/srep46450	342	Angel Cruz-Roa	2017	Article	Sci Rep-Uk
https://doi.org/10.1016/j.neucom.2016.01.034	336	Jun Xu	2016	Article	Neurocomputing
https://doi.org/10.1097/PAS.0000000000001151	317	David F. Steiner	2018	Review	Am J Surg Pathol
https://doi.org/10.1117/12.2043872	310	Angel Cruz-Roa	2014	Proceedings Paper	Proc SPIE (2014)
https://doi.org/10.1109/ICPR.2016.7900002	294	Neslihan Bayramoglu	2016	Proceedings Paper	Proc ICPR (2016)
https://doi.org/10.1002/cac2.12012	286	Yahui Jiang	2020	Review	Cancer Commun
https://doi.org/10.1111/joim.13030	267	B. Acs	2020	Review	J Intern Med
https://doi.org/10.1016/j.eswa.2018.09.049	260	P. J. Sudharshan	2019	Article	Expert Syst Appl
https://doi.org/10.1038/s41551-020-0578-x	250	Bryan He	2020	Article	Nat Biomed Eng
https://doi.org/10.1016/j.ebiom.2017.12.026	242	Pegah Khosravi	2018	Article	Ebiomedicine
https://doi.org/10.1038/s41523-018-0079-1	219	Heather D. Couture	2018	Article	Npj Breast Cancer

Regarding references, the top 20 most frequently cited works are listed in Table 5. The most cited reference is “A Dataset for Breast Cancer Histopathological Image Classification” by Fabio Spanhol, published in *IEEE Transactions on Biomedical Engineering*, followed by Kaiming He’s “Deep Residual Learning for Image Recognition” and Karen Simonyan’s “Very Deep Convolutional Networks for Large-Scale Image Recognition”. As presented in Fig. 5B, these three articles are also the most prominent in the co-citation network, with 251, 235, and 147 citations, respectively.

**Table 5:** Overview of the top 20 references with the most citations

DOIs	Times cited^*^	First Author	Year	Type	Sources
https://doi.org/10.1109/tbme.2015.2496264	251	Fabio Spanhol	2016	Article	Ieee T Bio-Med Eng
https://doi.org/10.1109/cvpr.2016.90	235	Kaiming He	2016	Proceeding Paper	Proc CVPR IEEE (2016)
https://doi.org/10.48550/arxiv.1409.1556	147	Karen Simonyan	2015	Article	Arvix
https://doi.org/10.1145/3065386	147	Alex Krizhevsky	2017	Article	Commun Acm
https://doi.org/10.1001/jama.2017.14585	146	Babak Ehteshami Bejnordi	2017	Article	Jama-J Am Med Assoc
https://doi.org/10.1109/ijcnn.2016.7727519	142	Fabio Spanhol	2016	Proceeding Paper	Proc IJCNN IEEE (2016)
https://doi.org/10.1007/978-3-319-24574-4_28	137	Olaf Ronneberger	2015	Proceeding Paper	LNCS (MICCAI 2015)
https://doi.org/10.1038/s41598-017-04075-z	117	Zhongyi Han	2017	Article	Sci Rep-Uk
https://doi.org/10.1109/cvpr.2017.243	114	Gao Huang	2017	Proceeding Paper	Proc CVPR IEEE (2017)
https://doi.org/10.1371/journal.pone.0177544	114	Teresa Araújo	2017	Article	Plos One
https://doi.org/10.1109/cvprw.2009.5206848	105	Jia Deng	2009	Proceeding Paper	Proc CVPR IEEE (2009)
https://doi.org/10.1109/isbi.2009.5193250	101	Marc Macenko	2009	Proceeding Paper	Proc ISBI (2009)
https://doi.org/10.1007/978-3-642-40763-5_51	98	Dan C. Cireşan	2013	Proceeding Paper	LNCS (MICCAI 2013)
https://doi.org/10.3322/caac.21660	97	Hyuna Sung	2021	Article	Ca-Cancer J Clin
https://doi.org/10.1038/s41591-018-0177-5	94	Nicolas Coudray	2018	Article	Nat Med
https://doi.org/10.1038/s41591-019-0508-1	93	Gabriele Campanella	2019	Article	Nat Med
https://doi.org/10.1016/j.media.2019.05.010	92	Guilherme Aresta	2019	Article	Med Image Anal
https://doi.org/10.1109/tbme.2014.2303852	91	Mitko Veta	2014	Review	IEEE Trans Biomed Eng
https://doi.org/10.4103/2153-3539.186902	89	Andrew Janowczyk	2016	Article	J Pathol Inform
https://doi.org/10.1109/rbme.2009.2034865	87	Metin N Gurcan	2009	Review	IEEE Rev Biomed Eng

* Citation counts represent the combined total of self-citations and citations by others

The citation trajectory analysis illustrates the citation patterns of the top 10 references from 2014 to 2025 (Fig. 5C). Most publications show a rapid citation growth beginning in 2017, with peak citation counts concentrated between 2022 and 2024. Some articles maintain consistently high citation levels, reflecting sustained influence, while others exhibit a clear decline. This pattern highlights variations in the academic impact across publications and may indicate shifts in research focus or the emergence of new topics in recent years. By analyzing the co-cited references across different time periods, the results indicate that early research foundation was dominated by traditional machine learning focusing on conventional pathology images. Over time, research that achieved breakthrough results with convolutional neural networks in specific detection and classification tasks emerged as the new reference core, marking the rise of deep learning as the mainstream. In recent years, emerging high-impact literature has shown a focus on more complex model architectures and multi-instance learning frameworks applicable to whole slide images (WSIs), reflecting a shift in research towards handling more complex and comprehensive analysis tasks.

Figure 5D illustrates the research disciplines in AIBCP. The analysis shows that research papers are mainly published in journals related to “Mathematics, Systems, Mathematical,” “Molecular, Biology, Immunology,” and “Medicine, Medical, Clinical.” Most citations, in turn, come from journals in “Systems, Computing, Computer”, “Molecular, Biology, Genetics”, and “Health, Nursing, Medicine”, indicating the interdisciplinary feature of AIBCP.

### Analysis of keywords

In this section, we analyzed keywords related to AIBCP. The most frequent keyword is “breast cancer” (*n* = 586), followed by “deep learning” (*n* = 396), “digital pathology” (*n* = 177), “artificial intelligence” (*n* = 174), and “convolutional neural network” (*n* = 164). These technology-related terms highlight the strong intersection between medical research and computer science in this field. Commonly used techniques include “machine learning” (*n* = 115), “transfer learning” (*n* = 70), “feature extraction” (*n* = 55), and “neural network” (*n* = 49), underscoring the central role of neural network-based methods in AIBCP research. Furthermore, we found that “histopathological images” appeared 122 times, while “whole slide image” was mentioned 72 times, indicating that most studies are based on pathological imaging. Additionally, the keywords “diagnosis” (*n* = 98), “segmentation” (*n* = 68), “mitosis detection” (*n* = 51), “image analysis” (*n* = 48), and “tumor-infiltrating lymphocytes” (*n* = 45) reflect parameters relevant to the clinical and pathological diagnosis of breast cancer.

**Fig. 6:**
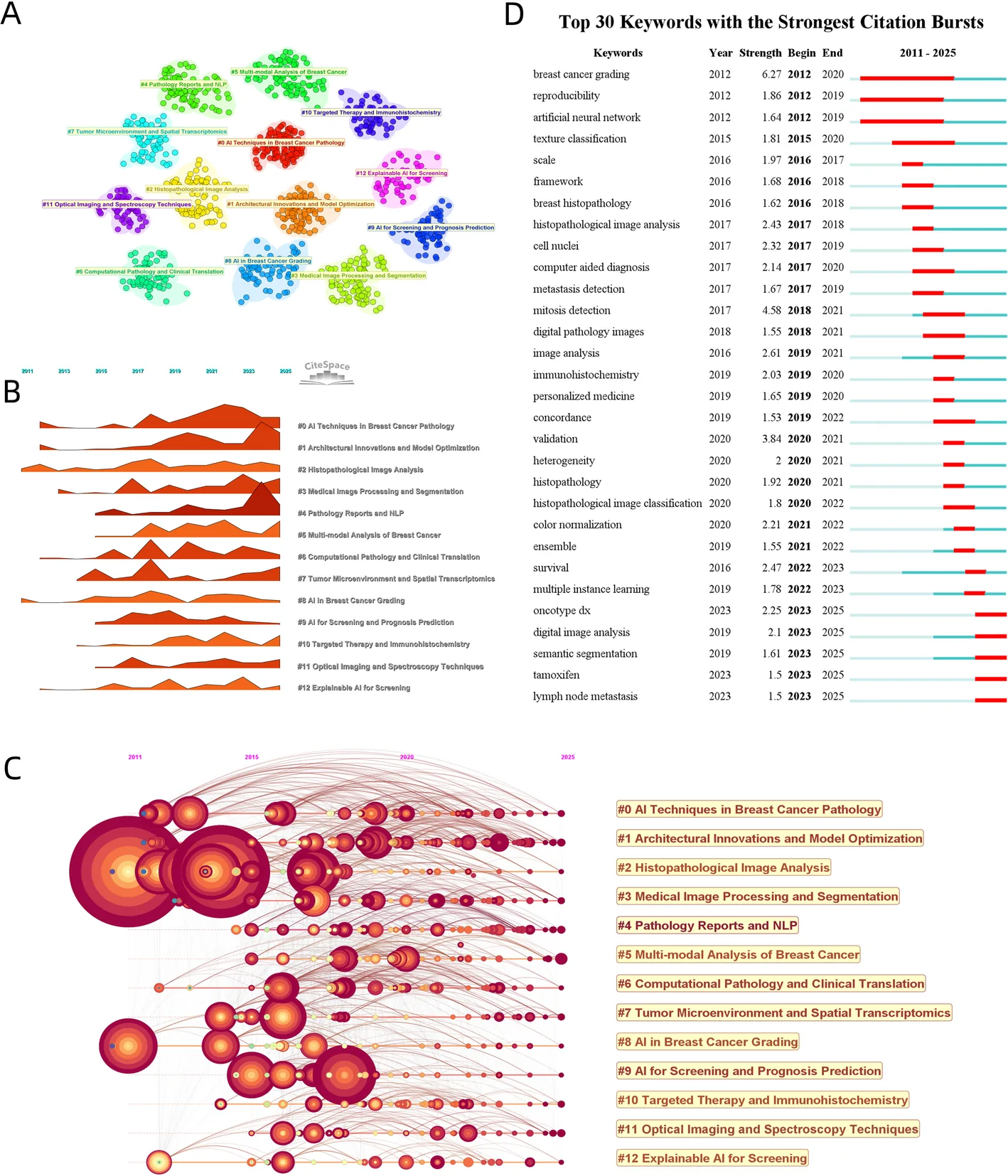
Visualization of Keywords Analysis. **A** Keyword clustering diagram. Each node represents a keyword, and distinct color clusters correspond to different thematic research groups. **B** Landscape view of thematic temporal trends, in which time is shown along the horizontal direction and the peaks reflect the evolution of thematic popularity over time. **C** Timeline view of keyword clusters, with nodes denoting keywords and connecting lines indicating keyword co-occurrence relationships. **D** Top 30 keywords with the strongest citation bursts, listing keywords that have experienced significant citation surges with their burst years and strength. Red segments in the timeline bars on the right show the duration of the bursts, reflecting dynamic shifts in research hotspots

As presented in Fig. 6A, the keywords were grouped into 13 distinct clusters. Based on the CiteSpace timeline and landscape map (Fig. 6B and C), this study reveals the evolution of research hotspots in the field of AIBCP. From 2011 to 2025, topics such as #0 (AI Technology for Breast Cancer Pathology) and #1 (Architecture Innovation and Model Optimization) have remained consistently active. Notably, #4 (Pathology Reports and Natural Language Processing (NLP)) and #12 (Interpretable AI for Screening) have shown significant growth in recent years (2023–2025), reflecting that natural language processing and interpretable AI have become emerging focal points. The rise of #7 (Tumor Microenvironment and Spatial Transcriptomics) highlights the deepening integration of multi-omics with pathology. Regarding the knowledge associations, the node size and link density in the knowledge graph show that #0 (AI Technology for Breast Cancer Pathology) serves as a core hub, with strong associations to topics like #2 (Histopathological Image Analysis) and #3 (Medical Image Processing and Segmentation), indicating a deep fusion of AI technology with traditional pathological analysis. The extension of node #6 (Computational Pathology and Clinical Translation) and its link to #9 (Screening and Prognostic Prediction) reveal the driving force of clinical translation needs. Furthermore, the distinct cluster of node #11 (Optical Imaging and Spectroscopy Techniques) suggests a specialized exploration within breast cancer imaging.

Figure 6D illustrates the top 30 keywords based on citation analysis using CiteSpace. The figure highlights periods during which the citation frequency of keywords, experienced significant surges. Among the top 30 keywords, citation surges for “breast cancer grading,” “reproducibility,” and “artificial neural network” occurred earlier, whereas “oncotype dx,” “digital image analysis,” and “semantic segmentation” showed a later surge.

### Analysis of abstracts

In this study, the BERTopic pipeline analyzed the abstracts of 1089 documents, identifying one noise topic (Topic − 1, *n* = 171) and 14 distinct thematic topics (Topics 0 to 13). As shown in Table 6, the largest one, Topic 0 (*n* = 583, 53.54%), highlights the significance of pathological image analysis in breast cancer pathology practice with topic words of “convolutional”, “deep learning”, and “histopathological”. Topic 1 (*n* = 157, 14.42%) reflects the AI applications in breast cancer pathology. Topic 2 (*n* = 38, 3.49%) focuses on mitosis detection and analysis, while Topic 3 (*n* = 26, 2.39%) centers on AI in clinical pathology, and Topic 4 (*n* = 25, 2.30%) emphasizes tumor microenvironment and prognosis. Although the remaining topics have less literature, they illustrate the expansion of AIBCP research into various academic directions, such as spatial transcriptomics and gene expression (Topic 9), optical diagnostic techniques (Topic 10, 11 and 12), multimodal and explainable AI Models (Topic 6), and NLP for pathology report analysis (Topic 5). This reflects the transition in research within this field from unimodal to multimodal pathomics.

**Table 6:** The results of topic analysis

Topic ID	Human-defined topic label	Count (%)	Topic Coherence Score (c_v)	Topic interpretability score
Topic 0	Deep Learning in Pathology Image Analysis	583 (53.54%)	0.4663	11.00
Topic 1	AI Applications in Breast Cancer Pathology	157 (14.42%)	0.6398	12.33
Topic 2	Mitosis Detection and Analysis	38 (3.49%)	0.8223	15.67
Topic 3	Artificial Intelligence in Clinical Pathology	26 (2.39%)	0.8587	16.33
Topic 4	Tumor Microenvironment and Prognosis	25 (2.30%)	0.2842	10.00
Topic 5	NLP for Pathology Report Analysis	18 (1.65%)	0.5155	11.67
Topic 6	Multimodal and Explainable AI Models	14 (1.29%)	0.8710	16.67
Topic 7	Crowdsourcing and Noisy Data Annotation	13 (1.19%)	0.7673	15.33
Topic 8	GANs for Image Generation and Enhancement	10 (0.92%)	0.2781	9.33
Topic 9	Spatial Transcriptomics and Gene Expression	9 (0.83%)	1.0000	18.00
Topic 10	Infrared and Optical Imaging Techniques	7 (0.64%)	0.4125	15.33
Topic 11	Optical Coherence Tomography	7 (0.64%)	1.0000	18.33
Topic 12	Raman Spectroscopy for Tissue Analysis	6 (0.55%)	0.9900	18.33
Topic 13	Demographic Bias and Molecular Biomarkers	5 (0.46%)	0.4578	13.33

In terms of topic quality, the topics exhibit significant differences. Topic coherence scores range from 0.2781 to 1.0000, with a total score of 0.6688. Notably, the coherence scores for Topic 6, 9, 11, and 12 are all above 0.87, indicating exceptionally high internal semantic coherence. In contrast, the coherence scores for Topic 4 and 8 are below 0.3, suggesting weaker internal semantic associations within their keyword sets. The thematic lexical overlap diversity score is 0.9714, and the semantic diversity score is 0.7683. This indicates that the literature demonstrates excellent richness and balanced distribution of core thematic keywords. However, the differentiation in semantic connotations is relatively limited, suggesting that the related research may exhibit a certain degree of thematic concentration or conceptual overlap. Topic interpretability was independently rated by three experts (Supplementary Table 5). A positive correlation trend is observed between interpretability scores and topic coherence. Topic 11, 12, and 9 achieved high scores in both coherence and interpretability. Overall, although the internal coherence of some smaller topics shows fluctuations, all topics were judged by experts to be clearly interpretable, validating the effectiveness of the topic modeling method used in this study.

**Fig. 7:**
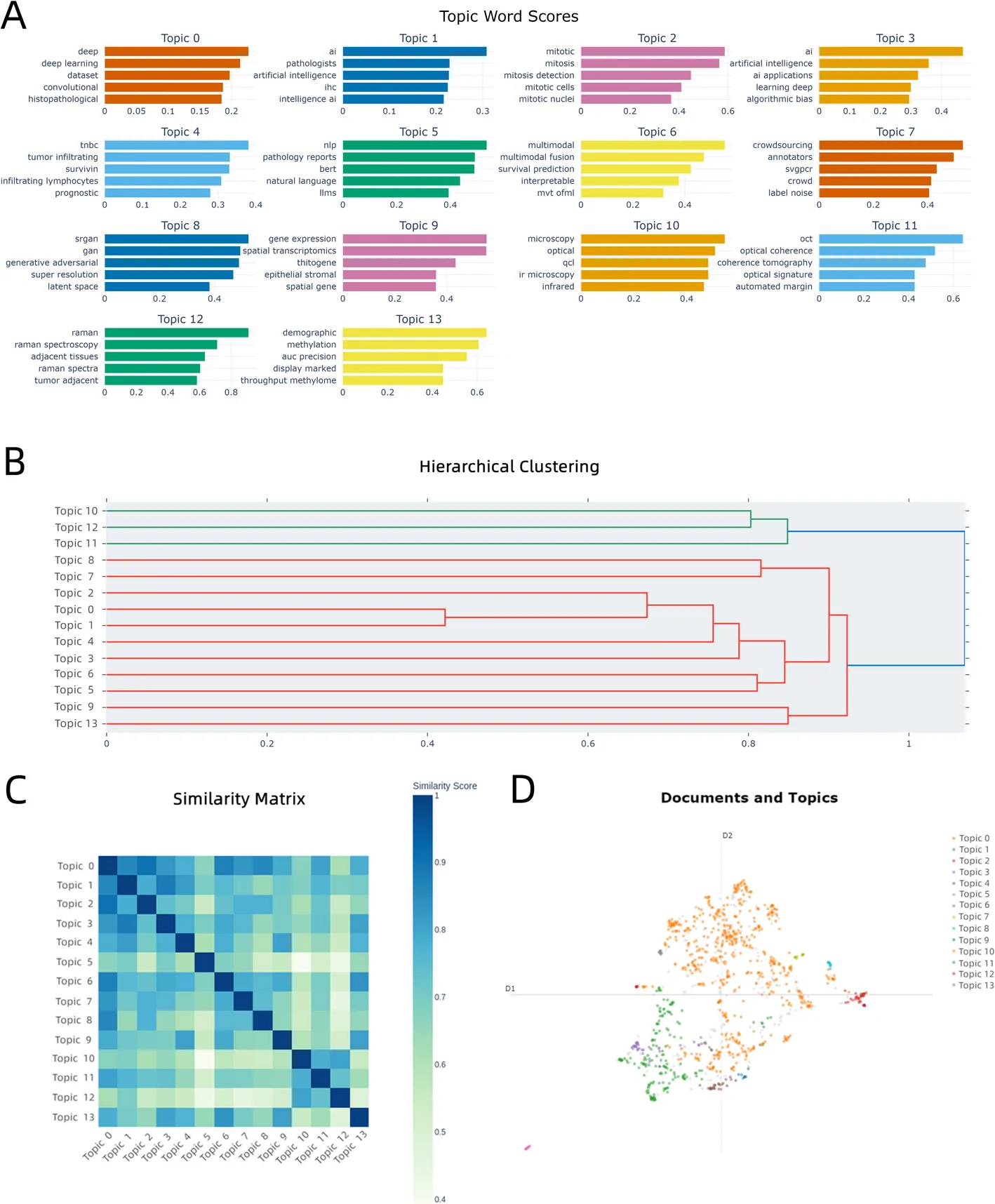
BERTopic Visualization Results. **A** Top five words per topic, sorted by topic word scores and displayed as horizontal bar charts, reflecting the core semantics of each topic. **B** Hierarchical clustering dendrogram of topics, connecting the 14 topics via branches of varying lengths, where shorter branches indicate higher semantic similarity between topics. **C** Topic similarity matrix heatmap. Color intensity represents the similarity between topic pairs, with darker blue indicating higher similarity. **D** Document-topic scatter plot, where each point represents a document, and its color corresponds to the dominant topic of the document, revealing spatial distribution and clustering of topics

Figure 7A illustrates Topics 0 to 13 along with their top five representative words ranked by topic word scores, reflecting the core research themes and focuses within AIBCP. Figure 7B provides a visual analysis of the 14 topics based on hierarchical clustering. The results indicate that Topic 10, 11 and 12 form independent branches characterized by high similarity, showing distinct features of AIBCP related to optical imaging and spectroscopy techniques as compared to other topics. According to the topic similarity heatmap analysis (as shown in Fig. 7C), varying degrees of correlation are evident among the 14 topics. The dark blue blocks along the diagonal represent the intra-topic self-similarity, while the light blue/green off-diagonal blocks indicate the inter-topic similarity, suggesting that although there is some semantic overlap among topics, their overall discriminability remains good. The document–topic projection plot (Fig. 7D) showcases the diversity of research hotspots in pathomics, with Topic 0 encompassing the largest number of documents, which reflects its preeminent position in computational breast pathology research. Additionally, with the exception of Topic 5, the remaining topics demonstrate concentration, likely due to their shared emphasis on pathological image studies.

Furthermore, the topic model constructed based on WOS was validated on the Scopus database (Supplementary Table 6 and Supplementary Fig. 1). Both databases identified 14 topics, with a high level of consistency in their core structures. The largest topic in both cases was “Deep Learning in Pathological Image Analysis,” accounting for 57.11% of the literature in Scopus and 53.54% in WOS, confirming the dominance of deep learning in this field. Other major topics, such as “AI Applications in Breast Cancer Pathology,” also consistently appeared with similar rankings, indicating that the model successfully captured the stable research structure of the field. The trends in topic quality assessment results were also comparable. The overall topic coherence score for Scopus was 0.5895. Several high-robustness topics performed prominently in both databases. For example, “Raman Spectroscopy-Based Tissue Analysis” achieved a perfect coherence score (c_v = 1.000) in both. The average topic interpretability scores were comparable, and the topics with the lowest scores overlapped between the two databases. This demonstrates that the model did not merely fit randomly but accurately identified cohesive, core research directions that exist across databases.

## Discussion

Although existing bibliometric studies have depicted the landscape of AI in oncology [29–33], they were unable to reveal the specific development pathways of AIBCP. To address this gap, we conducted a bibliometric analysis of 1089 publications related to AIBCP. The analysis considered publication year, countries and regions, authors, journals, citations, keywords and abstracts. Similar to bibliometric analyses in other fields [34, 35], it provides a foundational understanding of the field’s current development and applications, highlighting influential articles and authors, and outlining the potential directions for future research innovation. Compared with bibliometric studies on AI in general oncology, the focus of this study is more precise. For example, the oncology AI analysis by Koçak et al. covers a wide range of topics [30], while our study is deeply centered on breast cancer pathology with a particular emphasis on WSI analysis and deep learning models. In contrast to the bibliometric analysis on AI in oncology radiomics by Zhang et al. [32], our study highlights the distinctive features of digital pathology in terms of data and methodologies. Furthermore, different from the bibliometric analysis concerning the application of AI in oncology care [33], our findings reveal that current AIBCP research remains heavily concentrated on diagnosis and prognosis prediction, with insufficient attention paid to multimodal fusion and prospective clinical workflow evaluation. Through the above comparisons, this study clarifies the AIBCP field from the disciplinary pattern as a highly specialized frontier branch that is in the critical stage from technical validation to clinical integration.

Our bibliometric analysis shows that the annual increase of publications in the field of AIBCP demonstrates a consistent growth trend, especially after the year of 2017. This growth is driven by multiple factors. Technologically, deep convolutional neural networks provided a new paradigm for pathological image analysis, while advancements in GPU computing power and the widespread adoption of open-source frameworks like TensorFlow and PyTorch lowered the technical barriers. From a policy perspective, AI and precision medicine have become strategic priorities for many nations. Clinically, the WSI technology has enabled the digitization of pathological images, and the release of high-quality public datasets has addressed the data bottleneck for model training, making large-scale, reproducible research possible.

Although breast cancer has a high incidence worldwide, the primary research hubs in this field are the USA, the European Union, China, and India. These countries and regions typically have significant advantages such as research funding, high-performance computing clusters, the coverage of digital pathology equipment, and the reserve of high-level scientific talent. In contrast, many developing countries and regions, constrained by shortages in these critical resources and infrastructure, have not been able to fully realize their research potential. This landscape may lead to multiple effects. On the one hand, algorithmic models developed based on data from these countries/regions may have reduced generalizability when applied to areas with different healthcare conditions. On the other hand, the global distribution of research outcomes and benefits from clinical translation may become further imbalanced. To address this challenge, we recommend that future efforts should focus on establishing open, user-friendly algorithm platforms and toolkits, promoting the collaborative sharing of de-identified, multi-center, and multi-regional clinical datasets, and facilitating the cross-border flow of knowledge, data, and computational resources through international collaborative projects. Furthermore, countries with higher PPMP, such as Sweden and the Netherlands, typically have highly concentrated scientific resources, mature innovation mechanisms, and a high proportion of researchers, thereby achieving exceptional per capita scientific efficiency in AIBCP field. In contrast, despite the vast total volume of publications from countries like China and India, their lower number of PPMP (< 0.2 PPMP) reveals that their publications primarily rely on the large populations rather than high individual productivity. These findings reflect that intensive research environments are more conducive to the generation of knowledge and innovation in the field of AIBCP.

The analysis of author and institutional collaboration networks indicates that high-impact research typically emerges from close interdisciplinary and inter-institutional collaboration. Core teams composed of pathologists and AI experts can ensure that algorithm development directly addresses the key challenges in breast pathology. The expertise of pathologists is crucial for defining clinically meaningful morphological features, guiding the model’s focus, and providing interpretable explanations, which directly determines whether the tool can ultimately be trusted by pathologists. Regarding model validation and clinical promotion, multi-center collaborations that yield externally validated datasets with pathological diversity are essential for assessing a model’s generalizability. Therefore, open collaborative networks can lower the barriers to translating laboratory research into deployable clinical tools by integrating diverse expertise and data resources.

Keyword analysis highlights the diverse research topics within AIBCP from multiple perspectives. In terms of technology, prominent keywords include “convolutional neural network,” “transfer learning,” and “neural network,” indicating that deep learning, particularly neural networks, plays a central role in AI applications for breast cancer pathology. Additionally, the keyword “whole slide image” suggests that most studies utilize WSI rather than traditional pixel images. This preference is due to WSI’s ability to provide a comprehensive view of pathology, capturing both cellular details and overall tissue structure [36, 37]. Many studies have applied deep learning to WSIs for tasks such as histopathological image classification, biomarker analysis, prognosis prediction, and multimodal research [38–40]. Key application-related keywords identified in the literature include “classification,” “segmentation,” “mitosis detection,” “neoadjuvant chemotherapy,” “prediction,” and “breast cancer grading.” These keywords reflect the main clinical applications of breast pathology at present, highlighting that current research mainly focuses on addressing the critical challenges and complexities encountered in clinical pathological diagnosis [41].

BERTopic analysis of abstracts identified 14 distinct topics, outlining the current sub-directions of research in this field. Topic 0, comprising more than 50% of the documents, underscores that deep learning based digital pathology image analysis remains the core focus and technical foundation. This finding arises, firstly, because image classification and segmentation tasks align precisely with the inherent advantage of deep learning, making WSI analysis the most natural and technically mature research entry point in this field. Secondly, the most influential public datasets in the field primarily provide data in the form of images. This data format implicitly reinforces image analysis as the predominant research paradigm. Finally, unlike focusing solely on image analysis, clinical workflow integration involves complex hospital information systems, ethical considerations, and physician work habits, leading to longer research cycles. Consequently, research dedicated to image analysis is more readily published. The emergence of smaller topics, such as Topic 5 and 9, indicates that the research area is actively attempting to integrate multidimensional data, including molecular and clinical texts. Topic 6 addresses the “black box” challenge posed by multimodal and complex models in AI research for breast pathology. Finally, Topic 10, 11, and 12 form independent branches, demonstrating that the integration of optical imaging and spectroscopy techniques with AI holds unique significance in breast pathology research. This illustrates how the field is actively intersecting with disciplines such as biophysics to explore new growth points.

In the BERTopic analysis, 15.70% of the documents were categorized as “noise”. The abstracts of documents under Topic − 1 are broad in content, frequently using general terms such as “artificial intelligence”, “machine learning”, and “medicine”, and lack specific information pointing to particular technical methods, clinical problems, or biomarkers. The BERTopic model, based on the current parameter setting for minimum cluster size, was unable to clearly assign them to any topic with well-defined boundaries. A few document abstracts are extremely brief, failing to provide sufficient semantic information for the model to make effective distinctions. We believe it is reasonable to cautiously handle these “noise” documents during the analysis. Forcibly including literature with vague topic boundaries or insufficient information would instead reduce the interpretability of the topic model. Although these documents were excluded, our core conclusions remain unchanged, as verified by the results from the Scopus data. Furthermore, the existence of “noise” topics actually highlights the importance of text cleaning preprocessing and optimizing model parameters to adapt to highly interdisciplinary fields in future research. However, from another perspective, the “noise” group still contains certain research directions that have not yet formed a mainstream consensus but hold significant potential. Among these, several studies point towards data privacy, security, and computational infrastructure, highlighting that ethical and engineering challenges have become unavoidable issues in promoting the deployment of AIBCP. It does not directly address pathological diagnosis problems, but it resolves the ethical and engineering bottlenecks in the application of AIBCP.

Despite the exponential growth in scholarly publications within the field of AIBCP, this vigorous academic activity has not yet translated into mature clinical products or widespread routine application. This gap has become a critical bottleneck in the advancement of the AIBCP domain, and the reasons for it are multifaceted. First, although performance of AI model continues to achieve breakthroughs on closed datasets, its clinical deployment still faces significant challenges. The “black box” nature of AI models undermines the trust of pathologists, while interpretability is a fundamental requirement for clinical decision-making. Additionally, the robustness of algorithms is often constrained by the source of training data. Variations in tissue processing, staining protocols, and scanning equipment across different medical institutions can lead to significant declines in model performance, hindering widespread adoption. Second, the majority of existing studies remain at a low level of evidence-based medicine. In the field of AIBCP, the vast majority are retrospective, single-center studies focused on algorithm development and validation. Prospective, multicenter clinical trials capable of demonstrating that AI tools improve patient outcomes, diagnostic efficiency, or cost-effectiveness within actual clinical workflows are still exceedingly rare. Finally, a successful clinical AI tool must be a solution that seamlessly integrates into existing pathology department information systems. Currently, many studies focus on optimizing the model itself but overlook human-computer interaction and the resulting workflow changes. This oversight may lead to the rejection of even technically viable tools in clinical practice.

However, our study has several limitations. First, we used WOS as the only source for collecting bibliometric data. However, the database has limited coverage of important conference proceedings in computer science and artificial intelligence. This may result in insufficient representation of cutting-edge algorithmic and technological advances in the findings, while relatively overestimating clinical application research, which is more prominent in biomedical journals. Therefore, the conclusions of this study primarily reflect the research landscape at the level of publications in high-quality journals, rather than the full picture of its technological development. Furthermore, due to the limitations of the WOS data format, we were unable to effectively exclude author self-citations from the analysis. Therefore, the presented citation networks and impact metrics include contributions from self-citations, which to some extent affects the assessment of the research’s external impact. However, these differences are still within a certain range, and most of the key conclusions of this study are still consistent with other studies [42–44]. Second, the interpretability of bibliometric analysis is influenced by terminology heterogeneity, differences in citation motivations, and inconsistencies in author keywords within the raw data. This may result in certain emerging topics being obscured or the overemphasis of mature topics due to concentrated terminology. Therefore, the research trends identified in this study should be interpreted as a macro-directional indicator reflecting the mainstream research forces, rather than a precise depiction of all nuanced branches within the field. Finally, given the rapid development of the AIBCP field and the typical time lag between the production of academic literature and its indexing in databases, the research hotspots, technological clusters, and development trends captured in this study based on data from a specific time point primarily reflect the current research landscape as evidenced by visible literature up to the time of data collection. Disruptive technologies that are still in their infancy and have not yet gained substantial representation in the academic literature may not be fully reflected in the results of this study [45].

Finally, we present our recommendations for future studies. First, while the AI system demonstrates comparable accuracy to that of pathological experts in pathological image analysis, the underlying principles of its image analysis remain unknown [46–48]. Clarifying the decision-making principles of AI in pathological image analysis should be a key research objective in AIBCP field. At the same time, enhancing interpretability can provide a foundation for medical scientists and legal professionals to better understand the analysis results produced by AI [49–51]. Recently, Naas et al. proposed a breast cancer classification model based on deep vision transformer and introduced multiple explainability methods to highlight the most important features in the model’s predictions. This research strategy not only helps reveal the decision-making mechanism of the model but also enhances pathologists’ understanding of and trust in AI-generated outputs [52]. Second, current pure morphological analysis of WSI struggles to address the more complex needs of prognostic prediction and treatment decision. The biological behavior of tumors is fundamentally determined by a combination of their morphological appearance, molecular characteristics, and microenvironment [53]. Existing single-modal models, however, often treat tissue as a homogeneous entity, overlooking tumor heterogeneity at spatial and molecular levels [54], which directly limits the accuracy and generalizability of clinical predictions. As emerging topics like multimodality are gaining rapid attention, future breakthrough advances will arise from the deep integration of multimodal data. This involves leveraging WSIs to provide morphological information, utilizing genomics and transcriptomics data to elucidate molecular mechanisms, and integrating these with imaging data as well as clinical follow-up and treatment information, to construct algorithmic models capable of accurately predicting patient clinical outcomes. For example, a study developed a multilayer fusion model based on pathological images from axillary lymph node puncture and ultrasound images, and its performance in predicting breast cancer lymph node metastasis outperformed that of single-modality models [55]. In addition, novel techniques such as optical imaging and spectroscopy techniques provide label-free information on the tumor microenvironment, opening up entirely new data modalities for deep learning [56]. Finally, the current medical AI models are all examples of weak AI, thus it must rely on human supervision and correction [57]. However, current research in this field seems to focus on the development of AI algorithms themselves while seldom considering the role of humans in AI-based pathological image analysis or the improvement of humans’ effectiveness with AI. Encouragingly, research in other fields has already pointed the way. For instance, a study on cervical cytology screening designed an AI pre-screening and pathologist review workflow, demonstrating that this model maintains high sensitivity while significantly improving diagnostic efficiency and reducing inter-observer variability [58]. This indicates that human participation in AI has been proven to be effective in supplementing the shortcomings of AI diagnosis. It can be argued that, compared to an era dominated by complete AI, we are closer to an era that integrates both human and AI. Therefore, research on these interactions must be put on the agenda.

## Conclusions

In recent years, the use of AI in breast cancer pathology has grown rapidly. However, the increasing volume of annual publications makes it challenging for researchers and clinicians to gain a concise understanding of the field. To address this, we conducted a bibliometric study to provide a comprehensive overview of AIBCP. While deep learning based pathological image analysis remains the core focus, the field is increasingly expanding into multimodal and clinical research. Key future directions include improving AI interpretability, advancing multimodal studies, and optimizing framework for human-AI collaboration. We hope these findings will serve as a valuable resource for researchers and pathologists, guiding the continued advancement of AI-based breast cancer pathological diagnosis.

## Supplementary Information

Below is the link to the electronic supplementary material.


Supplementary Material 1.


## Data Availability

All data generated or analyzed during this study are included in this published article and its supplementary information.

## Abbreviations

AI: Artificial intelligence
AIBCP: Artificial intelligence in breast cancer pathology
WOS: Web of Science
PPMP: Publications per million population
WSI: Whole slide image
NLP: Natural language processing
