# Supplementary material for: Evolution of artificial intelligence in breast cancer pathology: a bibliometric analysis
Source: Discov Oncol. 2026 Jun 18;17:1262. doi: 10.1007/s12672-026-05388-0 (PMC13522322; doi:10.1007/s12672-026-05388-0)
Supplement: Supplementary file 1 [file 12672_2026_5388_MOESM1_ESM.pdf]

Supplementary Table 1 Completeness of the Data

Entries	Number of Docs	Completeness
**Sources**	882	80.99%
**Abstracts**	1089	100.00%
**Affiliation**	1089	100.00%
**Author(s)**	1089	100.00%
**DOI**	1056	96.97%
**Keywords-Authors**	936	85.95%
**Keywords-Plus**	774	71.07%
**References**	1088	99.91%
**Year**	1089	100.00%

Supplementary Table 2 BERTopic Parameter Sensitivity Analysis Report

Overview:

Number of parameter combinations tested: 50

Optimal quality score: 0.752

Average quality score: 0.556

Average number of topics: 14.5

Average noise ratio: 0.353

Parameter Space:

{ “n_neighbors”: [5, 10, 15, 20, 30],
“min_dist”: [0.0, 0.1, 0.2, 0.5],
“n_components”: [2, 3, 5, 7],
“min_cluster_size”: [3, 5, 7, 10],
“min_samples”: [1, 2, 3, 5],
“nr_topics”: [10, 15, 20, “auto”],
“diversity”: [0.1, 0.2, 0.3, 0.4, 0.5]}

Impact of Parameters on Quality Score:

min_dist: 0.297

n_neighbors: 0.205

min_samples: 0.154

nr_topics: 0.133

diversity: 0.108

min_cluster_size: 0.067

n_components: 0.057

Key Findings:

1. Most important parameter: min_dist
2. Average optimal n_neighbors: 13
3. Average optimal min_dist: 0.09
4. Range of topic numbers: 0 - 62

Recommended Configurations:

Comprehensive recommended combination: {“n_neighbors”: 15, “min_dist”: 0.1, “n_components”: 3, “min_cluster_size”: 5, “min_samples”: 1, “nr_topics”: 15, “diversity”: 0.3}

Supplementary Table 3 Overview of the top 20 countries/regions with the most publications

Countries/Regions	Publications	Citations	Publications per million population	Avg. pub. year	Avg. citations
**USA**	246	11119	0.754	2021.642	45.199
**China**	232	6156	0.167	2022.595	26.534
**India**	191	3036	0.140	2022.476	15.895
**United Kingdom**	94	2727	1.424	2021.904	29.011
**Canada**	54	854	1.456	2021.981	15.815
**South Korea**	48	1999	0.937	2022.125	41.646
**Saudi Arabia**	48	468	1.590	2023.708	9.750
**Sweden**	47	2292	4.671	2022.638	48.766
**Netherlands**	42	4000	2.436	2021.762	95.238
**Spain**	38	577	0.806	2022.395	15.184
**Pakistan**	37	642	0.163	2022.568	17.351
**Italy**	35	1264	0.586	2022.371	36.114
**Germany**	34	1734	0.413	2021.588	51.000
**Malaysia**	31	279	0.954	2022.742	9.000
**Australia**	25	793	1.012	2022.320	31.720
**Turkey**	23	753	0.285	2022.783	32.739
**Egypt**	22	211	0.211	2023.273	9.591
**Iran**	21	343	0.247	2022.952	16.333
**France**	20	1977	0.298	2022.000	98.850
**Japan**	19	445	0.150	2023.105	23.421

Supplementary Table 4 Overview of the top 20 institutions with the most publications

Institutions	Locations	Publications	Citations	Avg. Pub. Year	Avg. Citations
**University of Toronto**	Canada	22	323	2021.545	14.682
**Radboud University Nijmegen**	Netherlands	21	3152	2021.143	150.095
**Karolinska Institute**	Sweden	21	723	2023.381	34.429
**Emory University**	USA	20	412	2021.450	20.600
**University of Warwick**	United Kingdom	19	796	2021.474	41.895
**Harvard Medical School**	USA	18	609	2021.833	33.833
**Ohio State University**	USA	17	304	2023.529	17.882
**Case Western Reserve University**	USA	16	2964	2019.250	185.25
**University of Nottingham**	United Kingdom	13	660	2021.077	50.769
**Karolinska University Hospital**	Sweden	13	237	2023.538	18.230
**Sichuan University**	China	13	298	2022.308	22.923
**Universitair Medisch Centrum Utrecht**	Netherlands	12	2413	2022.333	201.083
**Tsinghua University**	China	12	656	2022.667	54.667
**Northwestern University**	USA	12	232	2021.583	19.333
**Sunnybrook Health Sciences Centre**	Canada	12	189	2021.667	15.750
**Princess Nourah bint Abdulrahman University**	Saudi Arabia	12	87	2023.583	7.250
**Universiti Malaya**	Malaysia	12	85	2022.667	7.083
**Memorial Sloan Kettering Cancer Center**	USA	11	1742	2022.273	158.364
**Linköping University**	Sweden	11	901	2021.364	81.909
**Stanford University**	USA	10	765	2022.500	76.500

Supplementary Table 5 The Interpretability Rating Scale

Question	Score		
Do the topic labels accurately summarize the research content?	Very accurate	5	
	Accurate	4	
	Neutral	3	
	Inaccurate	2	
	Very inaccurate	1	
Are the keywords highly relevant to the topics?	Very accurate	5	
	Accurate	4	
	Neutral	3	
	Inaccurate	2	
	Very inaccurate	1	
Is the topic distinct from other topics?	Very accurate	5	
	Accurate	4	
	Neutral	3	
	Inaccurate	2	
	Very inaccurate	1	
Is the example document consistent with the topic label?	Very accurate	5	
	Accurate	4	
	Neutral	3	
	Inaccurate	2	
	Very inaccurate	1	
Total Score			

Supplementary Table 6 Topic Analysis Results from Scopus Database

Topic ID	Human-defined Topic Label	Count (%)	Topic Coherence Score (c_v)	Topic Interpretability Score
**Topic 0**	Deep Learning in Pathology Image Analysis	1136 (57.11%)	0.4572	12.33
**Topic 1**	AI Applications in Breast Cancer Pathology	105 (5.28%)	0.7568	14.00
**Topic 2**	Traditional ML for Image Segmentation	101 (5.08%)	0.5775	12.67
**Topic 3**	AI Prediction of Treatment Response	61 (3.07%)	0.4631	11.67
**Topic 4**	Spatial Transcriptomics and Gene Expression	48 (2.41%)	0.4927	12.00
**Topic 5**	Biomarker and Receptor Status Analysis	41 (2.06%)	0.3842	10.00
**Topic 6**	NLP for Pathology Report Analysis	23 (1.16%)	0.4005	10.67
**Topic 7**	Multimodal and Explainable AI Models	9 (0.45%)	0.1893	9.33
**Topic 8**	Raman Spectroscopy for Tissue Analysis	8 (0.40%)	1.0000	17.33
**Topic 9**	Multiplex Imaging and Tumor Microenvironment	7 (0.35%)	0.4949	12.33
**Topic 10**	Crowdsourcing and Noisy Data Annotation	7 (0.35%)	0.9560	16.67
**Topic 11**	Active Learning for Efficient Annotation	5 (0.25%)	1.0000	17.67
**Topic 12**	Quantum-Inspired Computing for Classification	5 (0.25%)	0.7988	14.33
**Topic 13**	Infrared and Optical Imaging Techniques	5 (0.25%)	0.2824	10.33

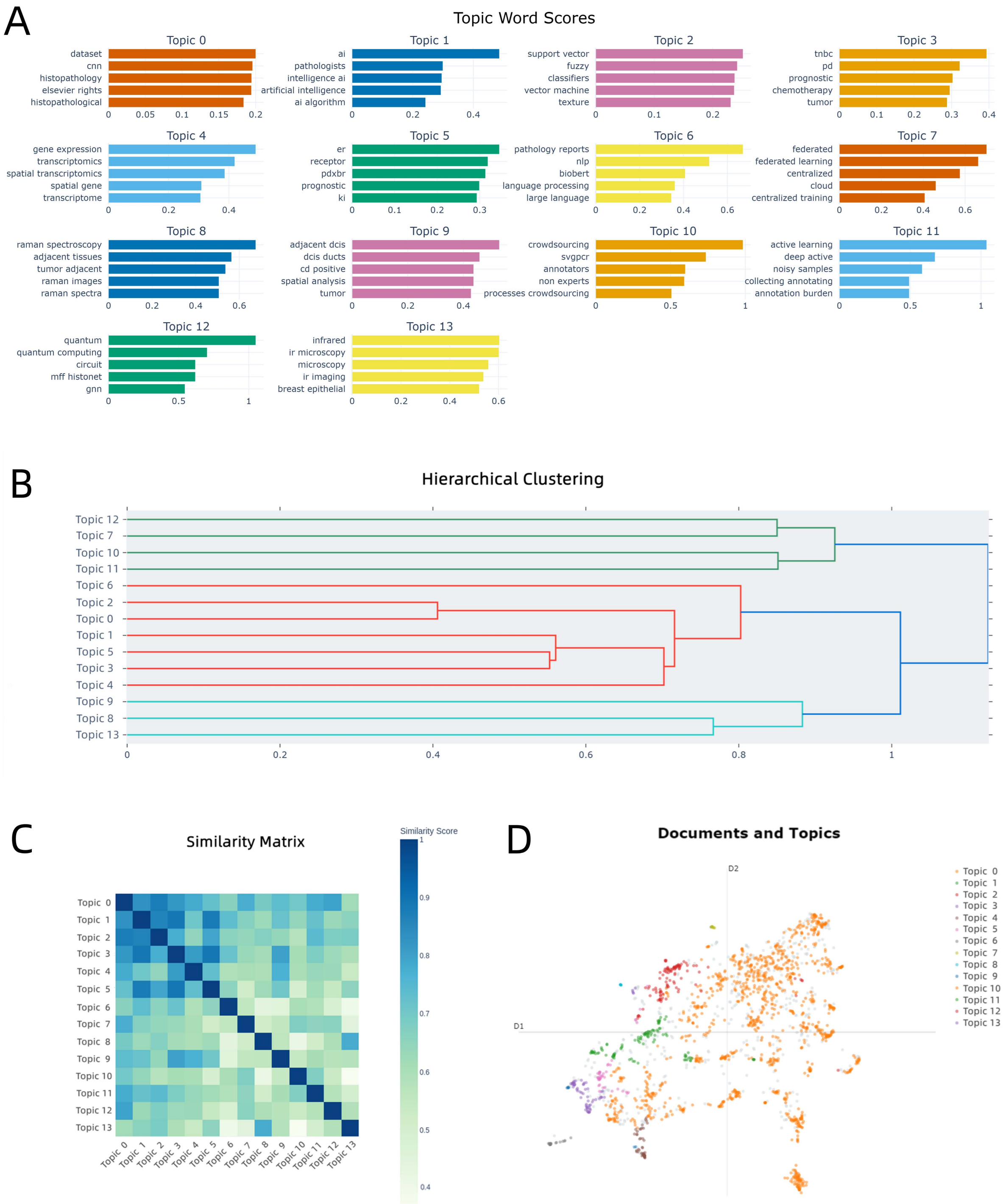

Supplementary Figure 1. BERTopic Visualization Results of Scopus Database. (A) Topic Word Weights: Horizontal bar charts show the top five keywords per topic. (B) Hierarchical Clustering: A dendrogram links the 14 topics; shorter branches denote higher similarity. (C) Topic Similarity Matrix: A heatmap shows pairwise similarities, with darker blue indicating higher similarity. (D) Document–Topic Projection: Documents are plotted as points, colored by their dominant topic.
